# Nutritional Optic Neuropathies: State of the Art and Emerging Evidences

**DOI:** 10.3390/nu12092653

**Published:** 2020-08-31

**Authors:** Matilde Roda, Natalie di Geronimo, Marco Pellegrini, Costantino Schiavi

**Affiliations:** Ophthalmology Unit, S. Orsola-Malpighi University Hospital, University of Bologna, 40138 Bologna, Italy; natalie.digeronimo@outlook.it (N.d.G.); marco.pellegrini@hotmail.it (M.P.); costantino.schiavi@unibo.it (C.S.)

**Keywords:** nutritional optic neuropathy, nutritional deficiencies, cobalamin, folic acid, thiamine, copper

## Abstract

Nutritional optic neuropathy is a cause of bilateral, symmetrical, and progressive visual impairment with loss of central visual acuity and contrast sensitivity, dyschromatopsia, and a central or centrocecal scotoma. The clinical features are not pathognomonic, since hereditary and toxic forms share similar signs and symptoms. It is becoming increasingly common due to the widespread of bariatric surgery and strict vegetarian or vegan diets, so even the scientific interest has recently increased. In particular, recent studies have focused on possible pathogenetic mechanisms, and on novel diagnostic and therapeutic strategies in order to prevent the onset, make a prompt diagnosis and an accurate nutritional supplementation, and to avoid irreversible optic nerve atrophy. Nowadays, there is clear evidence of the role of cobalamin, folic acid, thiamine, and copper, whereas further studies are needed to define the role of niacin, riboflavin, and pyridoxine. This review aims to summarize the etiology, diagnosis, and treatment of nutritional optic neuropathy, and it is addressed not only to ophthalmologists, but to all physicians who could come in contact with a patient with a possible nutritional optic neuropathy, being a fundamental multidisciplinary approach.

## 1. Introduction

Nutritional optic neuropathy is a possible, although rare, cause of visual loss. It is considered part of the metabolic neuropathy group, which includes heredodegenerative, toxic, and nutritional optic neuropathies [1]. All these conditions are characterized by mitochondrial metabolism impairment, which is congenital in the first category and acquired in the others. In case of nutritional neuropathies, damage to mitochondria is due to the lack of some crucial nutrients needed for their correct function, such as copper and group B vitamins, in particular B12 (cyanocobalamin), B1 (thiamine), and B9 (folic acid). These deficiencies cause an interruption of electron transport, and consequently a reduction of ATP production, leading to poor vitality of cells [1]. Some other mechanisms may explain the nerve damage associate with nutritional deficiencies, such as axon demyelination, in particular for what concerns B12 and folate deficiencies, and oxidative stress resulting from the lack of the antioxidant role of these nutrients.

What we know about the disease is principally due to epidemics that developed during periods of war and famine. Some examples are Strachan’s syndrome in Jamaican sugar cane workers in the 1880s, and optic neuropathy in prisoners of the Japanese during World War II, tropical amblyopia in Nigerians, Cuban epidemic optic neuropathy, and Tanzanian epidemic optic neuropathy [2]. The Cuban epidemic of optic neuropathy was fundamental in the understanding of the pathophysiologic mechanism of optic nerve damage. The similar clinical presentation of Leber hereditary optic neuropathy led to a further serological investigation that revealed how different environmental factors contribute to mitochondria damage and alteration of oxidative phosphorylation.

**Figure 1:**
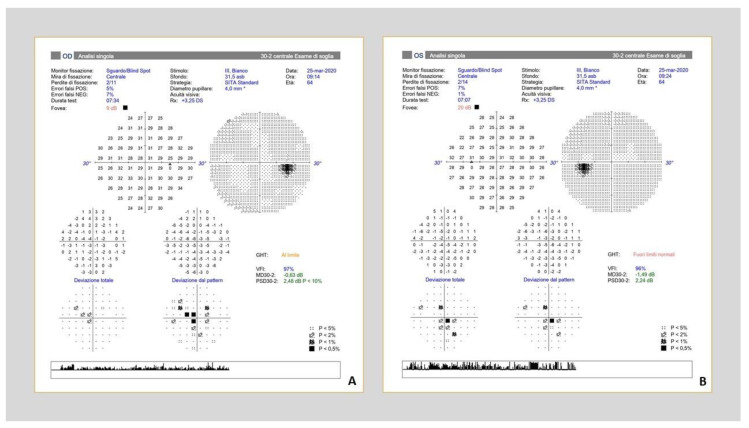
Bilateral central scotoma in a 62 y-o patient with nutritional optic neuropathy due to cobalamin deficiency. Note that the peripheral field is preserved. (**A**) Right eye; (**B**) Left eye.

Nutritional optic neuropathy usually presents with subacute, bilateral, fairly symmetrical, painless visual loss. Patients typically develop dyschromatopsia, loss of contrast sensitivity, and a central or centrocecal scotoma, whereas the peripheral field is mostly preserved (Figure 1). Most often, they do not present relatively afferent pupillary defect (RAPD), and in the first phase, optic disc has no clinical changes, but later in the disease, it can appear pale, swollen, or hyperaemic, and there is typically a preferentially loss of the papillomacular bundle fibres. A possible explanation of this phenomena is that small-caliber, parvocellular retinal ganglion cells located in the papillomacular bundle have more limited mitochondrial energy reserves if compared with the larger caliber, magnocellular cells, leading to a lower threshold to apoptosis of these small cells [3]. Optic atrophy may appear as the final stage of the disease [4], therefore it is important to identify the disease early and intervene as soon as possible to correct the deficiencies with the supplementation therapy, which has been proved to be the only effective treatment.

The deficiencies of vitamin B12, folic acid, thiamine, and copper are rare in developed countries, but they are becoming increasingly common due to the popularity of bariatric surgery [5] and vegan diet [6,7]. In low- and middle-income countries, inadequate food intake is the main cause of nutritional optic neuropathy, and it represents a worldwide health problem.

This review aims to resume the etiology, clinical features, diagnosis, and treatment of nutritional optic neuropathies, and we will discuss the clinical implications of new evidences and future research direction.

## 2. Etiology

Micronutrients known to be responsible for nutritional optic neuropathy are vitamin B12 (cobalamin), folic acid (vitamin B9), thiamine (vitamin B1), and copper, while there is no clear evidence of the role of niacin (vitamin B3), riboflavin (vitamin B2), and pyridoxine (vitamin B6) deficiencies [2]. Vitamins of the B group play crucial roles as coenzymes for enzymatic reactions in different biological systems, and vitamin B12, B6, and B1, the so-called neurotrophic vitamins, are essential for maintaining the health of the nervous system, including the optic nerve [8]. Multifactorial etiologies could coexist in some patients, and micronutrients deficiencies could be associated with alcohol and tobacco abuse [9].

### 2.1. Vitamin B12 (Cobalamin)

Vitamin B12, or cobalamin, is an essential water-soluble vitamin obtained through the ingestion of animal-sourced foods, such as meat, fish, and eggs, or fortified grain-based foods. In the stomach, it binds tightly to the intrinsic factor (IF) produced by gastric parietal cells, and the B12-IF complex is absorbed in the terminal ileum. In the bloodstream, it binds to transcobalamin II, and it is delivered to all the cells of the body, and to the liver where it is stored (Figure 1) [10]. Vitamin B12 is essential in lipid, carbohydrate, and protein metabolisms, in hematopoiesis, and in the conversion of 5-methyltetrahydrofolate to tetrahydrofolate, of homocysteine to methionine, and methylmalonic acid to succinyl coenzyme A [10]. The main cause of vitamin B12 deficiency is prolonged dietary deprivation in low-income countries, and a decreased bioavailability or interference with absorption, storage or transport in developed countries, first of all due to pernicious anemia, an autoimmune disease characterized by the atrophy of the gastric parietal cells.

The clinical presentation of B12-deficiency is insidious and includes haematological manifestations (pallor, dizziness, tachycardia, shortness of breath), gastrointestinal manifestations (weight loss, diarrhea) and neurological manifestations (paresthesia, ataxia, peripheral neuropathy). In particular, as a coenzyme in the methyl malonyl-CoA mutase reaction, vitamin B12 is crucial in myelin synthesis, and its deficiency leads to an incorporation of abnormal fatty acids into neuronal lipids, altering correct nerve transmission [11]. The optic neuropathy is caused by the degeneration of optic nerve fibers myelin lamellae and the damage of retinal ganglion cell axons. Recent studies [12,13,14] showed that cobalamin could act as an intracellular superoxide scavenger, underlining its importance as a neuroprotector in neuronal cells. Chan W. et al. [15] demonstrated in vivo that the B12-deficiency causes an increase of superoxide-induced cell death of retinal ganglion cells. The optic neuropathy may be the first symptom of cobalamin deficiency and may precede the hematologic disturbance [16].

### 2.2. Folic Acid (Vitamin B9)

Folic acid is a water-soluble vitamin of B group, which is found mostly in polyglutamate form in foods such as legumes, yeast, fruit, and leafy green vegetables. The oxidized monoglutamate form (folate) is rare in nature and is found in fortified foods and dietary supplements. Folic acid is hydrolyzed to the monoglutamate form before absorption, thanks to carboxypeptidase II (GCPII), which is primarily localized in the proximal part of the small bowel. Once inside cells, it is transformed once again into 5-methyl-THF and from here transported through the portal vein system into the circulation [17]. Derivatives of folate work as a coenzyme in several methylation reaction and are involved in pyrimidine and purine synthesis by providing one-carbon building blocks [18]. Folic acid deficiency is very uncommon in countries with mandatory or voluntary acid folic fortification programs, and most frequently, in developed countries, it is associated with nutritional deficiency, due to poor food intake and alcoholism, impaired absorption, and loss or increased requirements [19].

In adults, low folate status is associated with cognitive dysfunction in aging and can lead to serious consequences for the visual system. In fact, it is associated with nutritional amblyopia, pallor of the optic disc, disc atrophy and gradual decrease in vision. An important association is demonstrated between the lack of serum folate and the development of nutritional optic neuropathy. The deficiency leads to progressive visual loss, which usually appears with a painless bilateral, or occasionally unilateral, central scotoma and an alteration of color perception and dyschromatopsia, but without alteration of peripheral field [20].

The ways throughout folate deficiency may cause optic neuropathy are not fully understood yet. Several mechanisms have been studied and are likely to contribute to the damage. Reduction in folate concentration has been related to homocysteine accumulation, which would cause retinal neuron death and consequently an alteration of retinal layers, leading to maculopathy, but also an alteration of fiber layer, and therefore a damage to the optic nerve [21]. Furthermore, folate is an essential component of the enzyme tetrahydrofolate reductase (THFR) which is primarily involved in oligodendrocyte maturation. A lack of this nutrient leads to a downregulation of the enzyme with a consequent reduction in oligodendrocyte development, and thereby a lack of myelinization, which can affect optic nerve as well [22].

Moreover, folate deficiency seems to be related to an alteration of mitochondrial metabolism, due to a modification of the folate pathway which would involve the electron transport chain, and thereby reduce ATP production, in a similar way as it happens in Leber hereditary optic neuropathy [23]. Other hypotheses have been developed thanks to animal models: for example, folate concentration might be associated to macrophage-mediated inflammatory response [24], and also it could also affect the composition of extracellular matrix both in the retina and in lens [25].

### 2.3. Thiamine (Vitamin B1)

Thiamine, or vitamin B1, is a water-soluble B vitamin obtained through the ingestion of whole grains, meat, and fish, and which is absorbed in the gastrointestinal tract and stored in body tissues as thiamine diphosphate. Thiamine diphosphate participates in energy production as an essential cofactor for several enzymes in Krebs cycle, glycolysis, and pentose phosphate pathways [26]. Moreover, vitamin B1 has a role in nucleic acids, neurotransmitters and myelin synthesis, and has an antioxidative effect on nerve cells [8]. Thiamine deficiency leads to selective neuronal cell death by multiple mechanisms: cellular energy failure, focal lactic acidosis, blood-brain barrier breakdown, mitochondrial dysfunction and oxidative stress [27]. Most common risk factors correlated to thiamine deficiency are alcoholism, unbalanced nutrition, bariatric surgery, hyperthyroidism, and pregnancy [26]. The most frequent manifestation of thiamine deficiency is the Wernicke Korsakoff encephalopathy, which is characterized by the triad: acute confusional state, ataxia, and ophthalmoparesis [28]. Ocular features include nystagmus, ocular muscles palsy, conjugate-gaze palsies and, more rarely, anisocoria, light-near dissociation and optic neuropathy [29]. Optic neuropathy is a rare manifestation of B1 deficiency, and it is usually bilateral, severe, and associated with optic disc swelling [28,30,31,32]. Nevertheless, also peripapillary retinal nerve fibre layer (RNFL) infarcts with retinal hemorrhages in the absence of florid optic edema have also been described as a manifestation of optic neuropathy due to B1 deficiency [32,33,34]. The mechanism of thiamine deficiency optic neuropathy is still unknown, but seems to be principally due to the mitochondrial dysfunction: Bonhsack et al. postulated that mitochondrial dysfunction first gives rise to infarcts and hemorrhage of the RNFL and subsequent optic disc swelling appears only if mitochondrial damage is severe and prolonged [33].

### 2.4. Copper

Copper is a trace element, indispensable for organisms with oxidative metabolism. The total body copper content in adults is 100–150 mg and a normal individual has a 2–5 mg of copper daily requirement. It is absorbed primarily in the proximal small bowel, but gastric acid pH is fundamental to free copper from ingested food. It is an enzymatic cofactor involved in several tissues, such as hematopoietic, vascular, skeletal and especially nervous system, where it regulates the electron transport system, antioxidant defense, and iron metabolism [35].

Copper deficiency due to inadequate food intake is very rare. More frequently, it is related to malabsorption, in association with malabsorption syndromes including celiac disease, inflammatory bowel disease, and cystic fibrosis. Recently, complications caused by lack of copper are increased in consequence of partial or complete gastric resection, small bowel resections, or after malabsorptive bariatric surgical procedures.

Copper deficiency is related to hematologic abnormalities, while neurological manifestations are represented principally by myelopathy, peripheral neuropathy, myeloneuropathy, mononeuropathy, hyposmia, hypogeusia, and cognitive impairment [36]. Recently, optic neuropathy and visual loss have been reported as a consequence of copper deficiency as well. The optic neuropathy consequent to bariatric surgery is uncommon, and usually appears between 1,5 and 3 years after surgery, but it can develop even after 20 years, and it can be acute, subacute, or chronic.

The pathophysiologic mechanism that causes the optic nerve damage is not well known yet, but up to date the main hypothesis is that the copper deficiency would cause a demyelination, similarly as it happens in case of vitamin B12 deficiency. It might also be involved in mitochondrial metabolism impairment and oxidative stress responsible for a reduced vitality of neurologic tissue [37].

## 3. Diagnosis

**Figure 2:**
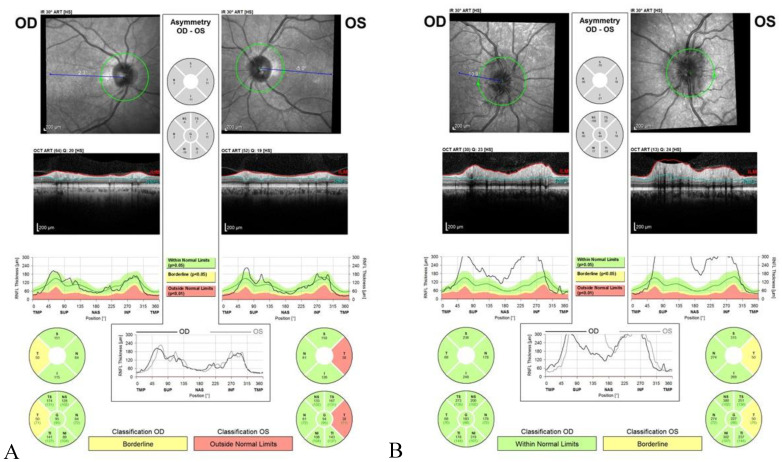
Optical coherence tomography showing (**A**) the thinning of temporal RNFL in a patient with nutritional optic neuropathy due to pernicious anemia (cobalamin deficiency); (**B**) optic disc swelling in a patient with Wernicke-Korsakoff encephalopathy (thiamine deficiency).

Nutritional optic neuropathy is characterized by progressive, bilateral, and symmetrical visual impairment with a loss of central visual acuity, centrocecal visual field defects, dyschromatopsia, and temporal optic disc pallor. Clinical features reflect the primarily papillomacular bundle affection, which is constituted by unmyelinated fibers susceptible to mitochondrial metabolism impairment [38,39]. Clinically, nutritional neuropathy is indistinguishable from other forms of heredodegenerative and toxic neuropathies [1], so an exhaustive patient’s history, including drugs/toxic exposure, familiar history, and medical history, must be taken, and prompt referral to an ophthalmologist is recommended. Clinical examination has to be done after the exclusion of a refractive error, using an appropriate refractive correction, and the presence of a maculopathy. Relative afferent pupillary defect (RAPD) is usually not present because the nutritional optic neuropathy is bilateral and symmetric. Fundus examination reveals a normal or hyperemic optic disc in early stages that becomes pale after months; in the thiamine deficiency, swelling of optic disc is typically present at the onset of the disease [40] (Table 1). A colour vision test, such as Ishihara test or Panel D 15 test, is necessary to rule out dyschromatopsia which is an early, and often the first, symptom of the disease; visual field (VF) shows symmetrical central or cecocentral scotoma (Figure 1), and visual-evoked potentials (VEP) reveal normal or near normal latency with significantly reduced amplitude; electroretinography (ERG) and optical coherence tomography (OCT) may be used to exclude a retinal disease. The use of optic nerve head OCT for diagnosis and monitoring nutritional optic neuropathies is still debated, but it may be helpful in detecting the thinning of RNFL before fundus changes appear. Thinning of RNFL begins in papillomacular bundle and then involves all the quadrants [41,42] (Figure 2). Imaging studies include MRI of the optic nerves, chiasm, and optic tract, which is usually performed to exclude a compressive or demyelinating lesion [43] (Table 2).

In the case of suspected nutritional optic neuropathy, it is recommended to perform a complete blood count to rule out anemia and macrocytosis, dosage of serum level of cobalamin, folate, thiamine, niacin, riboflavin, pyridoxine, serum copper (normal range: 70–125 µg/dL [44]) and ceruloplasmin levels, urinary copper dosage or in alternative plasmatic zinc level [45] (Table 3). In particular, the diagnosis of B12-deficiency should be supported by the measurement of serum cobalamin, methylmalonic and homocysteine levels. There is not a consensus of serum cobalamin level that defines the B12-deficiency, but most used cut-off is 150 pmol/L. If the serum cobalamin level is low (<150 pmol/L) or low-normal (150–399 pmol/L), the measurement of serum methylmalonic acid (cut-off range: 10 to 15 mol/L) and plasma homocysteine serum level (cut-off range: 271 to 376 nmol/L) is the next step: the increase of both markers confirms the B12-deficiency diagnosis [6]. In addition, it is recommended to measure serum levels of intrinsic factor and parietal cell antibodies to rule out pernicious anemia. Folate deficiency is determined by the evaluation of serum, plasma, or erythrocyte folate concentration, which does not detect acute changes in folate levels, but reflects the nutrient concentration over the lifespan of red cells. The diagnosis is primarily based on the presence of low serum or plasma levels (<3 ng/mL). Moreover, it cannot reliably distinguish between folate and B12 deficiency [46]. The dosage of serum methylmalonic acid and plasma homocysteine serum levels can help to distinguish the cobalamin-deficient from folate-deficient patients, most of whom display normal methylmalonic values [11,47]. Thiamine levels (normal range: 75–195 nmol/L [48]) can be evaluated in two ways: by assessing the degree of ThDP-saturation of a thiamine-dependent enzyme (erythrocyte transketolase assay), and by measuring thiamine metabolites in accessible tissues. The first one is considered more informative, as it demonstrates actual functionality of the vitamin. Thiamine esters and other metabolites are found in blood and urine [48].

Nevertheless, the diagnosis of nutritional optic neuropathy may be missed, or it could attribute to a unique deficiency when a multifactorial etiology is the responsible of the optic damage [49]. It could depend on local availability of tests, samples conservation and awareness of less common cause of nutritional optic neuropathy [49]. Moreover, it is always better to exclude heredodegenerative optic neuropathy testing the most common mutations of Leber’s neuropathy (m.3460G > A, m.11778G > A, and m.14484T > C) and autosomal dominant optic atrophy, because a micronutrient deficiency may be the trigger of the genetic optic neuropathy, since it causes a mitochondrial metabolism impairment [34]. In addition, it is important to exclude a toxic etiology, such as alcohol, tobacco, or drugs.

## 4. Differential Diagnosis

Nutritional optic neuropathies demand a differential diagnosis with several clinical conditions, and most of them are much more common than the nutritional neuropathies themselves. First of all, is basilar to draw up a thorough anamnesis, which needs to investigate patient’s age, concomitant illness, family history, exposure to drugs or toxins, visual impairment onset, ocular pain, and clinical features. Once the suspect of a neuropathy is established, is necessary to investigate the etiology of the disease. Differential diagnosis of neuropathy is essentially between inflammatory, infective, ischemic, toxic, and those related to systemic or hereditary illness. Inflammatory, ischemic, and infective forms are more often unilateral and associated with peculiar clinical conditions, that allow to exclude them from the differential diagnosis; while nutrition, toxic, and hereditary forms share the same clinical expression, and therefore need further investigation to be properly excluded.

### 4.1. Toxic Optic Neuropathies

These clinical conditions usually arise with a bilateral and painless visual loss, with subacute onset. Dyschromatopsia is one of the first symptoms, and patient typically report a loss of visual acuity of fixation point, followed by a progressive decline. This peculiarity is also showed by the visual field examination, which reveals a central or centrocecal scotoma, whereas the peripheral field is mostly preserved. In the early stages, the disc is normal or slightly hyperemic and swollen. Optic atrophy can develop after a variable interval. Pupillary reflexes are normal [9]. Some of the most common substances involved in the etiology are drugs, for instance cyclosporine, ethambutol, tacrolimus, and amiodarone, or toxins, such as methanol, ethylene glycol, tobacco, and nitrosoureas.

Ethambutol optic neuropathy is the most common toxic optic neuropathy, and it can lead to blindness if not detected early. It impairs oxidative phosphorylation and mitochondrial function by interfering with iron-containing complex I and copper-containing complex IV, leading to a cell damage similar to what happens during nutritional neuropathies. There is a well-known dose-response effect, in particular a dose of 60 mg/kg/d to 100 mg/kg/d is associated with a 50% risk of development of neuropathy, 5–6% of probability with a dose of 25 mg/kg/d and 1% with a dose below 15 mg/kg/die [50].

Between toxins, methanol is one of the major causes of optic neuropathy, because of the relatively easy accidental ingestion of this colorless and odorless liquid, that is often added to ethyl alcohol. These patients present with nausea, abdominal pain, metabolic acidosis, and a severe and bilateral visual loss. Optic disk is usually hyperemic with blurred margins, and the lack of pupillary reactivity to light suggests a poor prognosis [38]. Treatment is based on gastric lavage, dialysis, treatment of acidosis, and antidotes such as ethanol and fomepizole. Visual recovery is possible if the intervention is very early.

### 4.2. Hereditary Optic Neuropathies

Hereditary optic neuropathies are another group of pathologies that can be easily misdiagnosed with the nutritional neuropathies. Indeed, they are characterized by a bilateral visual loss, that occur with different timing and peculiarity depending on the mutation responsible for the disease. There are principally two hereditary forms: Leber hereditary optic neuropathy (LHON) and dominant optic atrophy (DOA).

LHON is a maternally inherited mitochondrial disease, which involves mostly young men, with age onset between 15 and 35 years old. It arises with a subacute visual loss in one eye, followed by the other eye in a few weeks, resulting in optic nerve atrophy and central visual loss. At presentation, optic disk often presents hyperemia, telangiectasias, and a swollen nerve fiber layer [51]. It is caused by a mitochondrial DNA mutation, and the most common mutation is 11778G > A/ND4, accounting for 70% of all LHON cases worldwide, while other known mutations are 3460G > A/ND1 and 14484T > C/ND6. These mutations lead to a dysfunction of the electron transport chain, therefore to a lack of energy production and increase of oxidative stress, leading to nerve cell death, in a similar way to what happens during toxic or nutritional neuropathies [52].

Dominant optic atrophy is the most common form of hereditary optic neuropathy with a prevalence of 1:12,000 to 1:50,000. Age of presentation is lower than LHON, involving patients in the first or second decade of life. In a similar way, it shows up with a progressive painless bilateral visual loss, central or centrocecal scotoma, dyschromatopsia involving primarily the blue-yellow axis, and decreased thickness of retinal nerve fiber layer presenting with a pallor of the optic disk. Papillomacular bundle is preferentially involved [53]. The gene responsible for the 60–95% of cases is the Optic Atrophy 1, a nuclear gene that codes for a *dynamin-related guanisine triphosphatase* (GTPase) necessary for mitochondrial inner membrane fusion and maintenance of mitochondrial cristae architecture. Once again, the loss of this protein leads to mitochondrial dysfunction and cell apoptosis [54].

Visual prognosis in these diseases is generally very poor, mostly in case of Leber neuropathy, where final VA is usually <20/200 in both eyes. The time needed to reach the final stage of the disease, on the other hand, is very variable, and can range from one year to decades. Idebenone and gene therapies are gaining more and more interest as promising treatments in both Leber disease and dominant optic atrophy. Idebenone, in particular, is a short-chain analogue of ubiquinone that can reduce ROS production, thus potentially reducing oxidative cellular damage. Different studies showed an earlier onset of visual recovery in LHON patient treated compared to the untreated [55,56]. To date, there are more evidences of the efficacy of this treatment in LHON, rather than in DOA, but this strategy seems to be promising in this disease as well.

Gene therapy is another promising approach being the eye an immunologically privileged site, and target genes can be delivered directly to the RGCs via intravitreal injection with aid of vector. However, the double-layer inner membrane of mitochondria is a relatively impermeable barrier, thus it is difficult to find a vector which is effective enough to have a significant metabolic impact on mitochondrial function. Until now, different approaches have been attempted using adenovirus-associated virus (AAV) type 2, and they demonstrated encouraging [57,58,59].

## 5. Treatment

The mainstay of treatment of nutritional optic neuropathy is the supplementation therapy, which is demonstrated to be very effective. Thus, it is fundamental to identify which nutrients are deficient, remembering that many patients have multiple micronutrient deficiencies, and eventually recognize the presence of toxins. It is also important to consider and exclude any kind of hereditary neuropathy through the application of genetic tests. Once the deficiency has been identified, it is necessary to set up a proper supplementation therapy.

In case of vitamin B12 deficiency, it can be replaced through oral supplementation or intramuscular injection; this second method allows a more rapid improvement in patients with severe neurological symptoms. The injections are usually made in doses of 1 mg/day for 2 weeks, then 1 mg twice a week until improvement, followed by a maintenance therapy of 1 mg every 1–3 months [10]. A new approach is the use of cyanocobalamin nasal spray as maintenance therapy, which showed efficacy after injection therapy in adults with neurological symptoms related to vitamin B12 deficiency [60]. It is recommended a dose of 1 mg of oral vitamin B12 per day indefinitely for patients who underwent bariatric surgery [10]. In case of folic acid deficiency, oral supplementation is the main approach, and folate is usually administered in a dose of 5 mg/day until complete recovery. Severe thiamine deficiency is associated with Wernicke encephalopathy, and the supplementation regimen consists in 500 mg of thiamine hydrochloride administered intravenously three times a day for 2–3 days, followed by 250 mg of thiamine given intravenously or intramuscularly daily for 3–5 days, until there is an improvement of clinical signs [29]. There isn’t an official consensus for the treatment of the associated optic neuropathy yet, however it seems that dosage used for the treatment of Wernicke encephalopathy is effective in resolving the neuropathy as well [40]. In case of copper deficiency there aren’t defined protocols either, but most often an intravenously supplementation of copper sulfate (2.4 mg mixed in 100 mL normal saline infused over 4 h for 5 days) is associated to oral copper gluconate (2 mg every 6 h). Often, copper deficiency is associated with the lack of other nutrients, in which case is essential to identify them and provide to correct their plasmatic levels in the same way.

A multidisciplinary approach is necessary for the correct management of patients with a nutritional optic neuropathy including ophthalmology, gastroenterology, clinical psychology, clinical biochemistry, general practice, dietetics, and other specialists. Patients may be continuously followed-up every 1–2 weeks, extending to 4–6 weeks if there is an improvement, for the first period, and then every 6–12 months [2,20]. The prognosis of nutritional optic neuropathy depends on severity and on time elapsed between the first signs and the time of micronutrients supplementation [1,2,49]; in most of the cases, visual recovery is complete, but if the deficiency is chronic, the damage leads to optic atrophy.

## 6. Conclusions

Nutritional optic neuropathies are considered a rare cause of visual loss. However, due to the widespread of bariatric surgery and of strict vegetarian or vegan diets [6,7], the cases of nutritional optic neuropathy are increasing even in developed countries. The main knowledge about nutritional optic neuropathies comes from the many case reports published during the last decades, while the most recent studies focus on the etiopathogenesis of optic nerve damage related to the single micronutrient deficiency [13,15,22,27], and on novel diagnostic strategies, such as the possible role of OCT to study the thinning of RNFL before fundus changes appears [41]. Micronutrients deficiency, in particular cobalamin, folic acid, thiamine, and copper deficiencies, should be ruled out in every patient with bilateral, progressive, and symmetrical visual loss because an early diagnosis and a prompt therapy change the prognosis. Visual impairment leads to significant consequences on both patients and healthcare system, so micronutrient supplementation should be set in all patients at risk of developing nutritional optic neuropathy. As opposed to ischemic, inflammatory and infective optic neuropathies, nutritional forms are more often bilateral and symmetrical. However, toxic and hereditary etiologies must be ruled out, since they share the same clinical expression and sometimes may co-exist with nutritional forms. We believe that further studies are needed to better define both preventive therapy and treatment in case optic neuropathy that has already developed. Nutritional optic neuropathy is not only a competence of ophthalmologists, but a multidisciplinary approach is fundamental for the correct management of the condition.

## Figures and Tables

**Table 1:** Symptoms and signs of nutritional optic neuropathy.

Symptoms
Progressive, bilateral, and symmetrical visual impairment
Central or centrocecal scotoma
Dyschromatopsia
Loss of contrast sensitivity
**Signs**
No relative afferent pupillary defect (RAPD)
Normal or hyperaemic optic disc (early stages)—except for thiamine deficiency where it is swollen already at early stages
Temporal, then diffuse optic disc pallor (late stage)
Thinning of RNFL in papillomacular bundle (early stage)
Thinning of RNFL involves of all the quadrants (late stage)
Normal or near normal latency with significantly reduced amplitude of VEP

**Table 2:** Ophthalmologic tests to perform in case of suspected nutritional optic neuropathy.

Test	
Colour vision tests (Ishihara plates, Panel 15D test)	Dyschromatopsia
VF	Central or cecocentral scotoma
VEP	Reduced amplitude, normal or near normal latency
RNFL OCT	RNFL thinning
ERG, OCT	To exclude retinal disease
MRI	To exclude compressive or demyelinating diseases

VF: Visual field; VEP: visual-evoked potentials; RNFL: retinal nerve fibre layer; OCT: optical coherence tomography; ERG: electroretinography.

**Table 3:** Normal and pathologic blood values of micronutrients involved in the development of nutritional optic neuropathies.

	Normal	Pathologic
**Vitamin B12**	200–900 pmol/L	<150 pmol/L
**Folate**	3–20 ng/mL	<3 ng/mL
**Thiamine**	75–195 nmol/L	<75 nmol/L
**Copper**	70–125 μg/dL	<70 μg/dL
